# Production of monoclonal antibody against recombinant NS3 protein of bovine viral diarrhea virus (NADL strain)

**Published:** 2016-09-15

**Authors:** Masood Reza Seyfi Abad Shapouri, Maryam Ekhtelat, Masood Ghorbanpoor Najaf Abadi, Mohsen Lotfi, Mohamad Rashno

**Affiliations:** 1 *Department of Pathobiology, Faculty of Veterinary Medicine, Shahid Chamran University of Ahvaz, Ahvaz, Iran;*; 2 *Department of Pharmacognosy, Faculty of Pharmacy, Ahvaz Jundishapur University of Medical Sciences, Ahvaz, Iran;*; 3 *Department of Quality control, Razi Vaccine and Serum Research Institute, Karaj, Iran;*; 4 *Department of Immunology, Faculty of Medicine, Ahvaz Jundishapur University of Medical Sciences, Ahvaz, Iran.*

**Keywords:** Bovine viral diarrhea, Monoclonal antibody, NS3, Recombinant antigen

## Abstract

This study was conducted to investigate the prevalence of subclinical mastitis caused by *Staphylococcus* spp. in ewes in West-Azerbaijan province of Iran. Molecular characterization of isolated *Staphylococcus *spp*.* from diseased ewes were performed using polymerase chain reaction (PCR) followed by restriction fragment length polymorphism (RFLP) and DNA sequencing of glyceraldehyde-3-phosphate dehydrogenase (*gap*) gene. Also, antibiotic resistance of staphylococcal isolates against different antibiotics was investigated. A total number of 900 milk samples from 450 native ewes in their mid-lactation period were examined by the California mastitis test (CMT). The CMT positive samples were cultured and bacteria were isolated from 86 (9.50%) glands and 74 (16.40%) ewes. The prevalence of subclinical mastitis in the examined ewes was 16.40%. Microbiological analysis of milk samples revealed that 27 out of 74 sheep with subclinical mastitis were infected with *Staphylococcus* spp. Amplification of *gap* gene of 27 *Staphylococcus* isolates generated a single amplicon of 933 bp in size confirming that isolates were belonged to *Staphylococcus* genus. Digestion of PCR products by *Alu*I endonuclease generated different RFLP patterns for each species. Nucleotide sequencing of *gap* gene followed by phylogenetic analysis showed that the most dominant *Staphylococcus *species were *S. epidermidis*, *S. xylosus* and *S. chromogenes*. Staphylococcal isolates showed the highest resistance to penicillin and ampicillin. In conclusion,* Staphylococcus* species, except for the southern parts of the province, play an important role in the development of subclinical mastitis in sheep in West-Azerbaijan province of Iran. Also, chloramphenicol, ciprofloxacin and neomycin are the most effective antibiotics for treatment of this disease.

## Introduction

Bovine Viral Diarrhea (BVD) is an economically important disease of cattle with a worldwide distribution. The BVD is caused by bovine viral diarrhea virus (BVDV) which belongs to Pestivirus genus within the family of Flaviviridae.^1^ The BVDV is capable of producing a broad range of clinical signs, ranging from most often asymptomatic infection to severe acute disease with signs from the enteric, reproductive or respiratory organs. Bovine fetus infected with non-cytopathic biotype of BVDV between days 30 and 125 of gestation can develop immune tolerance against the virus and will be born persistently infected (PI) shedding the virus continuously.^2^ Diagnosis of BVD relies on laboratory-based detection of its viral causing agent (particularly for the identification of PI animals) or virus specific antibodies. The most common laboratory method for this purpose is enzyme-linked immunosorbent assay (ELISA).^3^ The most immunogenic proteins of BVDV,^4^ including Erns and E2 structural proteins and the non-structural NS3 protein have been prepared as recombinant proteins and applied to design ELISAs for the detection of specific antibodies in cattle sera.^5^ The NS3 is an 80 kDa (p80) protein which contains an N-terminal serine protease domain and a C-terminal RNA helicase.^6^ Production of NS3 is essential for the viral RNA replication and cytopathogenicity.^7^ This protein is also highly conserved among pestiviruses and induces a strong humoral immune response in cattle exposed to live BVDV either naturally or by vaccination.^8^ Therefore, it is a proper candidate antigen to detect antibodies against the virus in the sera of infected animals. For this purpose, NS3 and NS3-specific monoclonal antibodies (MAbs) were used to design ELISAs (indirect and competitive ELISA) for the detection of specific antibodies against the virus.^5^^, ^^9^^-^^11^ During the recent years, economic impact of BVDV infections has led a number of countries in Europe to start eradication or control programmes.^12^^,^^13^ In Iran, the prevalence of BVDV antibodies in adult cattle is around 25.0%.^14^^,^^15^ It is therefore desirable to have a rapid, sensitive and reliable means of identifying infected animals for control and eradication of BVD. Anti-NS3 MAbs were produced mainly following immunization with whole virus. The main objective of this study was to produce monoclonal antibody against recombinant NS3 antigen of BVDV that was produced in an efficient bacterial expression system to design a local competitive ELISA for detecting infected animals in future.

## Materials and Methods

Materials. SP2/0 murine myeloma cell line and Balb/c mice were obtained from Razi Vaccine and Serum Research Institute, Karaj, Iran. Hypoxanthine aminopterin thymidine (HAT), hypoxanthine thymidine (HT), RPMI 1640 medium and fetal bovine serum (FBS) were purchased from Gibco Laboratories (Grand Island, USA). Anti-mouse IgG proxidase and polyethylene glycol (PEG) were obtained from Sigma (St. Louis, USA). All chemicals were of analytical reagent grade quality.

Expression and purification of MBP-NS3 fusion protein. Production of recombinant MBP-NS3 protein in pMalc2x expression vector, under the control of the lac promoter in E. coli BL-21 strain had been previously produced in our laboratory.^16^ For expression of MBP-NS3 protein, a bacterial colony which had no mutation in the NS3 insert was selected and cultured in high volume of ampicillin embedded Luria-Bertani (LB) broth media (Merck, Darmstadt, Germany) containing 20 mM glucose, until the OD 600 reached to 0.5. Then, protein expression was induced by adding isopropyl-β-D-thio-galactoside (IPTG) (Cinnagen, Tehran, Iran) at a final concentration of 1 mM. After 4 hr incubation at 37 ˚C, expression of the recombinant MBP-NS3 protein was examined by SDS-polyacrylamide gel electro-phoresis (SDS-PAGE). To further analyze, expressed protein(s) were analyzed by Western blotting, using a BVDV antibody positive bovine serum (data not shown). After expression, the bacterial pellet resuspended in column buffer and sonicated to release the bacterial proteins. Purification of the expressed protein (MBP-NS3) from the supernatant of the sonicated bacteria was carried out on a column of maltose-affinity chromatography based amylose resin.^17^ In order to this matter in the first step, the purification performed based on MBP’s affinity to amylase. Then in the second step, the MBP-NS3 protein was detached from amylose resin by using 10 mM maltose solution as a competitor with amylase. Recombinant protein (MBP-NS3) in collected samples was examined by SDS-PAGE. Maltose binding protein (MBP) is a fusion partner of about 42 kDa (without express alpha fragment of beta galactosidase) encoded by pMAL-c2X plasmid vector at the N-terminus part of the recombinant proteins. MBP molecule plus alpha fragment of beta galactosidase with an approximate weight of 50 kDa is in bacteria containing only pMAL-c2X. Several studies have shown that MBP is a soluble protein and can even solubilize fused recombinant protein.^18^^,^^19^ As solubility of the recombinant protein was enhanced by MBP at the beginning part of the recombinant molecule, the purification processes were done without any need to treatment with chemical substances like urea.

Immunization. Balb/c mice are usually chosen as the source of immune spleen cells. For this purpose, Balb/c mice, 4 to 6 weeks old, were immunized by IP injection of 100 µg of purified MBP-NS3 on days 0, 15 and 34. First injection was with complete Freund's adjuvant (Razi Institute). The Second and third injections were performed by using incomplete Freund's adjuvant (Razi Institute) in order to stimulate a good immune response. The mice were tail-bled, and the serum was assayed for antibody activity by an indirect ELISA on day 45. Mice with highest titer of anti-NS3 antibodies by indirect ELISA were selected and three days before fusion, a booster injection of MBP-NS3 without adjuvant was performed and their spleens were removed for fusion.^20^^,^^21^

Preparation of myeloma cells and mouse feeder cells. SP2/0 murine myeloma cell line is a good fusion partner for immune spleen cells because of its good growth rate, the efficiency of hybridoma production after fusion and because it does not synthesize or secrete any immunoglobulin chains. About 1 × 10^7^ SP2/0 cells in logarithmic phase with viability more than 95.0% were used for fusion. Mouse peritoneal cells (feeder cells), most of which are macrophages, are an effective source of soluble growth factors for hybridoma cells. For preparation of feeder cells, adult Balb/c mice were sacrificed and 8 mL 0.34 M chilled sucrose solution injected intra-peritoneally, entering directly at base of the sternum and rest tip of needle over liver. After gently massage of the abdomen, the fluid was withdrawn and viable cells were counted and diluted with HAT medium to 1 × 10^5^ feeder cells per mL. This cell suspension was added to the 60 inner wells of 96-well plates, 24 hr before fusion.^21^

Cell fusion and detection of anti-NS3 secreting hybrids. For hybridoma production, 1×10^7^ SP2/0 myeloma cells in logarithmic phase were fused with 1 × 10^8^ spleen cells from the immunized mice using polyethylene glycol as the fusing agent. The cells in fusion mix were re-suspended in 35 mL HAT medium and incubated at least 30 min in CO_2_ (8.0%) incubator (INCOmed, Memmert, Schwabach, Germany) at 37 ˚C. Then, 100 µL of the fusion mixture distributed to the 60 inner wells included feeder cells of 96-well plates and incubated five days in CO_2_ incubator. After five days, 100 µL HAT medium was added to each well and replaced with fresh HAT medium every other day.^21^ Aminopterin in the HAT medium blocks the de novo pathway. Hence, unfused myeloma cells die, as they cannot produce nucleotides by salvage and also de novo pathway. Unfused B cells (spleen cells) die as they have a short lifespan. In this way, only the B cell-myeloma hybrids survive. These cells produce nucleotides by salvaging pathway and antibodies (a property of B cells) also are immortal (a property of myeloma cells).^21^Two weeks after fusion, until hybrid cells cover 10.0% to 50.0% surface area of wells, culture supernatants were screened by indirect ELISA. Enzyme immunoassay (Nunc, Roskilde, Denmark) plates were coated with 50 µL recombinant MBP-NS3 per well at a concentration of 2 µg mL^-1^ in coating buffer (0.5 M NaHO_3_/Na_2_CO_3_, pH 9.3) at 4˚C overnight (MBP-NS3 ELISA). Also recombinant MBP was coated concurrently and separately (MBP ELISA). The unspecific bindings were blocked with 0.05% (v/v) phosphate buffered saline Tween-20 (PBST) containing 5.0% skim milk powder (Merck, Darmstadt, Germany) for 2 hr at 37 ˚C and washed three times with PBST. The supernatant of hybridoma was incubated for 1 hr at 37 ˚C, with sera (1:2000 in PBST containing 2.0% skim milk) from non-immunized and immunized mice as negative and positive controls, respectively and also a supernatant of other hybridoma as negative control. Then, plates were washed three times with PBST. The bound antibodies were detected with Goat anti-mouse IgG proxidase diluted 1:2000 in PBST containing 2.0% skim milk buffer for 1 hr at 37 ˚C. Finally, tetra-methylbenzidine (TMB; Merck) peroxidase substrate solutionwas added and left for 10 min at room temperature in the dark. The reaction was stopped by the stop solution (1 M H_2_SO_4_; Merck) and the absorbance was read at 450 nm using ELISA reader (Dynatec-MR 5000; Ashford, United Kingdom). Those positive hybrids in MBP-NS3 ELISA which did not react with recombinant MBP in MBP ELISA were selected as positive.^21^

Isolation and cloning of hybridoma cells. Several cloning were carried out until more than 90.0% of the wells containing single clones were positive for antibody production that indicating the cells are identical and have the same origin. For this purpose, positive clones were expanded into 24-well plates containing feeder cell and were grown overnight in CO_2_ incubator. Then the positive hybrids were cloned by limiting dilution (8 cells per mL) in HT medium (HAT without aminopterin) and 100µL of the dilution distributed in 96-well plates. The single clones with the highest anti-NS3 antibody titers using indirect ELISA were subcloned at least three times until all sub-clone supernatants were positive for antibody production. After three times cloning, positive subclones were grown to larger volumes and frozen in liquid N2, as soon as possible, in FBS containing 10.0% of DMSO (Merck).^21^

Reactivity of MAbs to recombinant and natural antigen. The stability of antibody secretion in the positive clones after three times cloning was monitored by ELISA and the reactivity of the anti-NS3 MAbs to recombinant and natural NS3 was evaluated by Western blotting. At first, recombinant NS3 antigen was electrophoresed on 10.0% vertical SDS-polyacrylamide gel and the protein band was transferred to nitro-cellulose membrane. After transfer, the nitrocellulose membrane was cut into strips and blocked with PBST containing 5.0% skim milk for 2 hr at 37 ˚C and washed three times with PBST. The strips were then incubated for 1 hr with the supernatant of each positive hybridomas separately. Concurrent a super-natant of other hybridoma as negative control and also sera (1:2000) from non-immunized and immunized mice were placed as negative and positive controls, respectively. Finally, the strips were washed again as above and incubated for 1 hr with Goat anti-mouse IgG proxidase diluted 1:2000 in PBST containing 2.0% skim milk. After washing, the strips were developed by 4-chloro 1-naphtol (Sigma) and H_2_O_2_ as substrate.^21^ For reactivity of MAbs to natural NS3 antigen, BVD virus propagated in bovine turbinate cells and the culture medium was centrifuged and the precipitate was suspended in 200 μL of SDS-PAGE sample buffer and 200 μL PBS. Then, this sample as natural Ag with MBP and recombinant nucleoprotein of influenza virus as marker in separate well were electro-phoresed in a 10.0% SDS-PAGE. At the completion of electrophoresis, the gel related to markers was cut and stained with coomassie blue and the rest of gel was evaluated by Western blotting same as before.^21^

## Results

**Fig. 1:**
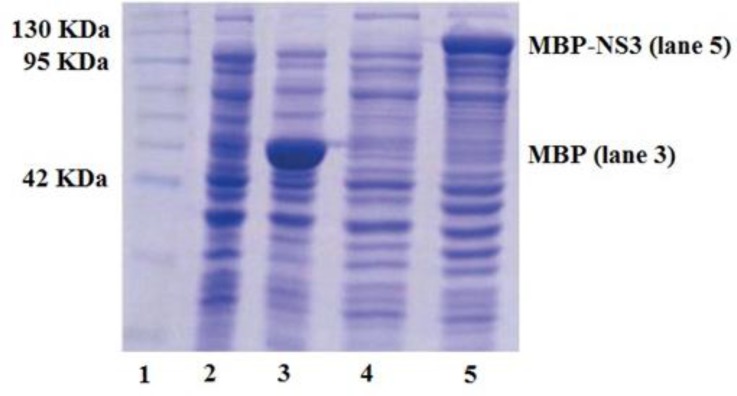
SDS-PAGE analysis of bacteria expressing MBP-NS3 fusion protein before (lane 4) and after (lane 5) induction by IPTG. The expression of a protein of about 117 kDa, corresponding to MBP-NS3 is shown in the lane 5. Lane 2 and 3 indicate a bacterium expressing MBP before and after induction by IPTG, respectively. The molecular weight marker is shown in Lane 1

**Fig. 2:**
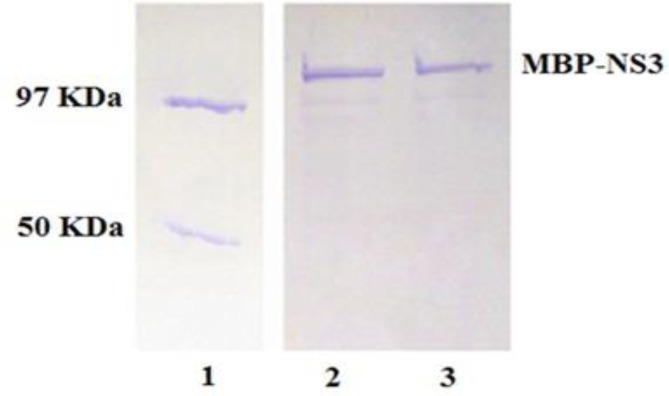
SDS-PAGE analysis of purified MBP-NS3 fusion protein by amylose-resin column. Lane 1 shows purified nucleo-protein of influenza virus (~97 KDa) and purified MBP (~50 KDa) from control colony containing only pMAL-c2X as protein weight marker. Lane 2 and 3 indicate purified preparation of MBP-NS3 fusion protein (~117 KDa

Expression and purification of MBP-NS3 fusion protein. Expression of recombinant MBP-NS3 protein in pMalc2x expression vector in E. coli BL-21 strain was induced by adding IPTG to a bacterial culture prepared as the instruction of pMalc2x expression system. Protein profiles of induced and non-induced bacteria by SDS-PAGE revealed that a polypeptide of about 117 kDa was expressed in the induced bacteria (Fig. 1). Considering molecular weights of 42 and 75 kDa for MBP and NS3 respectively, MBP-NS3 fusion protein was estimated to have an approximate molecular weight of 117 kDa. As shown in Figure 2, after purification of the expressed protein (MBP-NS3) on a column of amylose resin and analysis by SDS-PAGE, expression of the recombinant MBP-NS3 is clearly detectable.

Hybridoma production and isolation. After immunizing mice with the recombinant MBP-NS3, anti-NS3 antibody in serum of mice was determined by indirect ELISA on day 45. The OD values of immunized sera (1:2000 diluted) were from 1.40 to 1.49 compared with OD values of 0.09 in non-immunized serum (negative control). Spleen cells of the immunized mouse with highest titer of anti-NS3 antibody were fused with SP2/0 myeloma cells using PEG. Hybrids were selected by their ability to grow in HAT medium. Then, culture supernatants of primary clones were screened by MBP-NS3 ELISA and MBP ELISA. Those positive hybrids in MBP-NS3 ELISA which did not react in MBP ELISA were considered as positive. On the basis of primary screening, anti-NS3 antibody in the supernatant of 20 clones was identified with OD value from 0.20 to 1.10 (average 0.52). The positive clones with highest titer of anti-NS3 antibody were cloned and screened by indirect ELISA for three times. The OD values of ELISA were higher from 1.10 to 2.70 in second and third cloning with not reacting to MBP. However, anti-NS3 antibody production was stopped in three clones during cloning probably due somatic mutations or deletions of chromosome.

**Fig. 3:**
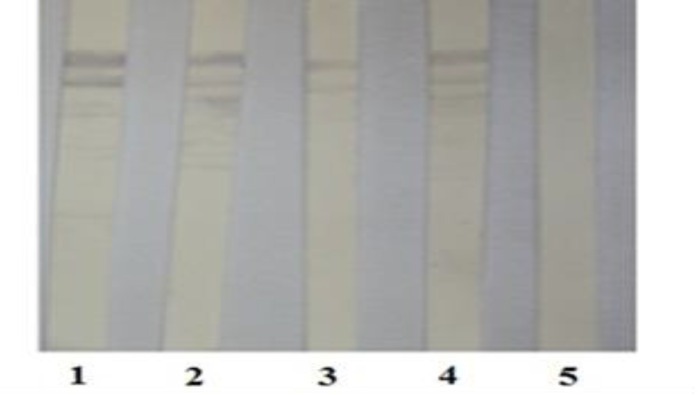
Reactivity of supernatants of subclones producing anti-NS3 MAbs to recombinant NS3 antigen by Western blotting. Lanes 1 and 5 belong to immunized and non-immunized mice sera as negative and positive controls, respectively. Lanes 2, 3 and 4 represent supernatant of subclones containing anti-NS3 MAbs

Reactivity of MAbs to recombinant and natural antigen. The positive clones after three times cloning were selected and the reactivity of the MAbs with recombinant NS3 antigen was screened and established by Western blotting (Fig. 3).

**Fig. 4:**
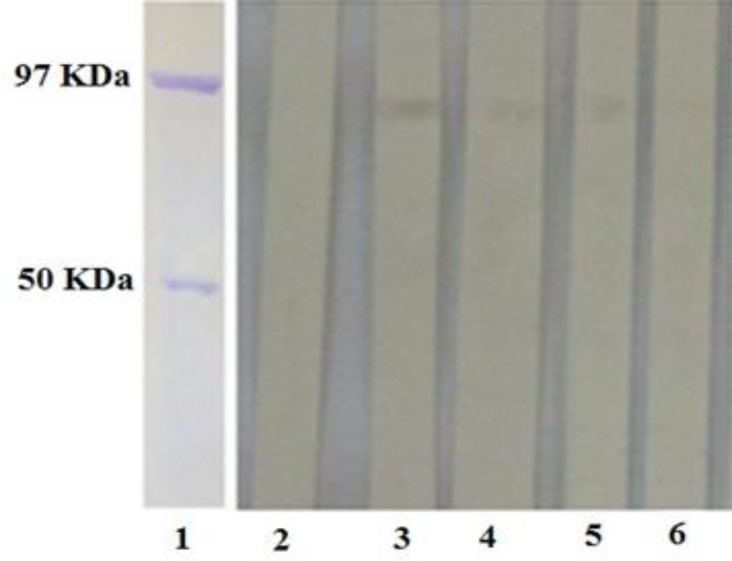
Reactivity of supernatants of subclones producing anti-NS3 MAbs to natural NS3 antigen by Western blotting. Lanes 1 shows purified nucleoprotein of influenza virus (~97 KDa) and purified MBP (~50 KDa) as protein weight marker. Lanes 2 indicates supernatant of an unrelated hybridoma. Lanes 3-6 represent supernatant of subclones containing anti-NS3 MAbs

Also the specificity and reactivity of the MAbs with the natural form of the NS3 antigen, as shown in Figure 4, were examined and confirmed with BVD virus infected cells as the source of the virus in Western blotting. In the Western Blot analysis, anti-NS3 MAbs reacted with a protein of BVDV that was lower than the 97 kDa marker, that were compatible with the approximate weight of natural NS3 (80 kDa) of BVDV.

## Discussion

Diagnosis of BVD relies on laboratory-based detection of its viral causing agent or virus specific antibodies. Identification of PI animals is carried out by virus isolation, RT-PCR and detection of viral antigens by immuno-histochemistry and AC-ELISA. Virus isolation and virus neutralization test (VNT), as the “gold standards” are sensitive and specific assays but cell culture dependent and labor intensive. ELISA is more suited for screening of large series of samples for detection of both antibodies and viral antigens.^22^^,^^23^ Bovine viral diarrhea virus non-structural protein 3 (NS3) has a critical role in the pathogenesis of BVDV. This nonstructural protein strongly induces humoral immune response and is highly conserved among pestiviruses.^8^^,^^24^ Therefore, several ELISAs have recently been developed to detect BVDV infections using recombinant NS3 protein and anti-NS3 MAbs.^5^^,^^9^^-^^11^^,^^25^ This protein has shown high sensitivity and specificity for detection of BVDV infection in comparison with whole virus antigen.^9^^,^^11^

After BVDV specific Mabs became available, several laboratory assays reported for BVDV detection based on anti BVDV MAbs. Several AC-ELISA were developed for rapid detection of BVDV based on either polyclonal and MAbs specific for one or more viral antigens following immunization with whole virus.^23^^,^^26^ In a research, an AC-ELISA were developed using MAbs against p125/p80 polypeptide of border disease virus (BDV) and BVDV for detecting virus antigens from the blood of infected cattle and sheep. This MAb ELISA was compared to existing ELISAs which rely on polyclonal antibodies (PAbs). The MAb detection ELISA was more sensitive than the PAb detection ELISAs.^27^ In a research, Mignon et al. developed an AC-ELISA using anti-gp48 and anti-NS3 MAbs for detecting BVDV antigens in blood samples with sensitivity and specificity100%. They proved the AC-ELISA was a good candidate for replacing virus isolation as a reference method for BVDV antigen detection in PI animals.^26^ In another research, 860 blood samples without antibodies to BVDV were examined in both virus isolation and in an AC-ELISA based on anti- p125/p80 MAbs. A total of 843 samples (98.0%) were positive in both tests, thereby showed this virus protein was highly conserved among different BVD viruses.^28^ Bedekovic et al. described an indirect immunofluorescence (IF) method based a pool of BVDV-specific monoclonal antibody (VLA, Weybridge, UK) using ear notch tissue samples for diagnosis of PI cattle. Compared with the RT-PCR assay, IF assay had a sensitivity and specificity of 100% and was a good alternative to RT-PCR and antigen ELISA with high speed and accuracy.^29^ The utilization of microparticle immuno-agglutination assay has been investigated using coated microparticles with anti-BVDV monoclonal antibodies for BVDV detection. The microparticle immunoagglutination was more sensitive than RT-PCR to detect the virus in the shortest time.^30^ Kameyama et al., developed an immunochromatographic test for rapid diagnosis of BVDV infections using anti-NS3 monoclonal antibodies of the virus. The sensitivity and specificity of this kit compared with virus isolation were 100% and 97.2%, respectively.^31^ Also an indirect immuno-peroxidase assay has been reported using anti-gp53 and anti-NS3 MAbs for detecting BVDV antigens in CNS.^32^ Lecomte et al., described a competitive ELISA using anti-P80 (NS3) MAbs that was more specific than an indirect assay for antibody detection of virus.^9^ In one study, a NS2-3 blocking ELISA was applied for detection of BVDV antibodies using 1189 milk per serum samples with sensitivity and specificity 96.9% and 97.8% respectively, compared with virus isolation.^11^ Bhatia et al. developed a competitive inhibition ELISA (CI-ELISA) for detection of antibodies against BVDV using prokaryotically expressed helicase domain of NS3 and an anti-NS3 monoclonal antibody. Their study proved that helicase domain of NS3 was equally useful as whole NS3 protein used in two commercial ELISA kits for detection of BVDV antibodies.^25^ The economic impact of BVDV infections has led a number of countries in Europe to start eradication or control programmes.^12^

To summarize, in the present study, for the first time, large amount of immunologically active recombinant NS3 protein in an expression system under the control of the strong promoter (lac) of vector pMalc2x were used to produce anti-NS3 MAbs by cell fusion assay. Anti-NS3 MAbs were screened by indirect ELISA and the reactivity of the MAbs with recombinant and natural antigen was established by Western blotting. Based on our results, it appeared that NS3 recombinant antigen and the specific monoclonal antibodies produced against it might be suitable for developing BVDV laboratory diagnostic assays especially competitive inhibition ELISA.
